# Critical dynamics and interictal epileptiform discharge: a comparative analysis with respect to tracking seizure risk cycles

**DOI:** 10.3389/fnetp.2024.1420217

**Published:** 2024-07-09

**Authors:** Amrit Kashyap, Paul Müller, Gadi Miron, Christian Meisel

**Affiliations:** ^1^ Computational Neurology, Department of Neurology, Charité–Universitätsmedizin Berlin, Berlin, Germany; ^2^ Berlin Institute of Health, Berlin, Germany; ^3^ NeuroCure Cluster of Excellence, Charité–Universitätsmedizin Berlin, Berlin, Germany; ^4^ Bernstein Center for Computational Neuroscience, Berlin, Germany; ^5^ Center for Stroke Research Berlin, Berlin, Germany

**Keywords:** epilepsy, iEEG, network, cycles, criticality

## Abstract

Epilepsy is characterized by recurrent, unprovoked seizures. Accurate prediction of seizure occurrence has long been a clinical goal since this would allow to optimize patient treatment, prevent injuries due to seizures, and alleviate the patient burden of unpredictability. Advances in implantable electroencephalographic (EEG) devices, allowing for long-term interictal EEG recordings, have facilitated major progress in this field. Recently, it has been discovered that interictal brain activity demonstrates circadian and multi-dien cycles that are strongly aligned, or phase locked, with seizure risk. Thus, cyclical brain activity patterns have been used to forecast seizures. However, in the effort to develop a clinically useful EEG based seizure forecasting system, challenges remain. Firstly, multiple EEG features demonstrate cyclical patterns, but it remains unclear which feature is best suited for predicting seizures. Secondly, the technology for long-term EEG recording is currently limited in both spatial and temporal sampling resolution. In this study, we compare five established EEG metrics:synchrony, spatial correlation, temporal correlation, signal variance which have been motivated from critical dynamics theory, and interictal epileptiform discharge (IED) which are a traditional marker of seizure propensity. We assess their effectiveness in detecting 24-h and seizure cycles as well as their robustness under spatial and temporal subsampling. Analyzing intracranial EEG data from 23 patients, we report that all examined features exhibit 24-h cycles. Spatial correlation, signal variance, and synchrony showed the highest phase locking with seizures, while IED rates were the lowest. Notably, spatial and temporal correlation were also found to be highly correlated to each other, as were signal variance and IED—suggesting some features may reflect similar aspects of cortical dynamics, whereas others provide complementary information. All features proved robust under subsampling, indicating that the dynamic properties of interictal activity evolve slowly and are not confined to specific brain regions. Our results may aid future translational research by assisting in design and testing of EEG based seizure forecasting systems.

## 1 Introduction

Epilepsy is a disorder of the central nervous system (CNS) characterized by the enduring predisposition to generate epileptic seizures. Means of better understanding and characterizing the risk of seizures, their onset, propagation and termination are therefore desirable Mormann et al. (2007). Approaching epilepsy as a network disorder in cortex has proven particularly promising in this regard Spencer (2002). What are needed, are metrics to characterize the evolving epileptic brain network on spatial and temporal scales. Such network-based metrics are particularly relevant for tracking changing seizure risk levels across 24-h and multi-day cycles where they may provide important insights into pre-seizure dynamics as well as success or failure of network-based seizure control and prevention Zaveri et al. (2020) Meisel and Loddenkemper (2020) Lehnertz et al. (2023).

Interictal epileptiform discharge (IEDs) are unique to epileptogenic tissue, and have long been used to characterize interictal activity clinically by helping localize seizure onset zones and track seizure risk periods Hufnagel et al. (2000) Barkmeier et al. (2012) Baud et al. (2018). Initially, IEDs were manually identified by EEG clinicians. Subsequently, automated detection methods were developed, which involved tracking the ratio between various band powers derived from iEEG data or, more recently, using machine learning methods. These approaches enabled continuous monitoring of IED frequency over extended periods of time Barkmeier et al. (2012) Gutierrez (2022) Quon et al. (2022).

Other network metrics to track epilepsy have been proposed from theory. One such theory in the setting of epilepsy as a network disorder relates to the notion that cortical network dynamics reside near a critical state at a boundary between distinct types of dynamics Meisel and Loddenkemper (2020). The transition to a seizure then constitutes a transition to another type of dynamics. Based on this framework, specific markers have been proposed to track cortex excitability and seizure risk, i.e., the distance to a critical point Meisel and Kuehn (2012) Meisel et al. (2015b) Maturana et al. (2020) Meisel (2020). A general feature of systems near a critical phase transition is critical slowing down Scheffer et al. (2009). Critical slowing down occurs because of the repeatedly slower recovery from small perturbations when a bifurcation or phase transition is approached Wissel (1984) Meisel and Kuehn (2012) Meisel et al. (2015a). Critical slowing down can be monitored by tracking a signal’s variance and autocorrelation, which, consequently, have been shown to contain information about a network’s state and seizure risk Meisel (2020) Maturana et al. (2020). Similarly, correlations in space are predicted by theory to peak at criticality, and can be captured by quantification of the decay of the spatial cross-correlation function Müller and Meisel (2023) or by quantifying phase synchronization across cortical sites Meisel et al. (2015b). Both spatial correlations measures have been shown to capture information on the excitability levels of cortical networks, such as under changing levels of antiseizure medication Müller and Meisel (2023) Meisel et al. (2015b). More recently, the study of seizure occurrence and interictal markers of seizure propensity has shifted from the order of minutes to hours to the order of days, months and even years, indicating the existence of certain cycles Meisel et al. (2015b) Baud et al. (2021) Proix et al. (2021) Maturana et al. (2020) Karoly et al. (2021). These changes have been linked to bodily rhythms suggesting that during certain phases of these cycles, the central nervous system is more susceptible to epileptic seizures.

Therefore, while different measures have been proposed to track the state of cortical networks and, specifically, seizure risk, it is not completely understood whether these different metrics all measure the same underlying dynamical changes. A better understanding of how different measures proposed as markers of seizure propensity are correlated, how strongly they exhibit cycles and how well these cycles correspond to seizure occurrence is therefore crucial to optimally tracking seizure risk. From a translational perspective, it is furthermore important to also understand how robust these metrics are under spatial and temporal subsampling. Implantable devices, which aim to reduce seizures through focal stimulation, often only have limited spatial electrode coverage and limitations in the amount of data they can sample over time Gutierrez (2022) Baud et al. (2018) Haneef et al. (2022). Therefore, the measures characterizing interictal activity have to be robust in tracking the cycles from shorter recordings, e.g., when the stimulation is turned off, as well as from fewer electrodes.

Here we study five measures related to seizure propensity: IEDs Hufnagel et al. (2000) Baud et al. (2018), variance and autocorrelation Meisel (2020) Maturana et al. (2020), as well as spatial correlation Müller and Meisel (2023) and network synchrony Meisel et al. (2015b). First, we characterize these measures in how strongly they exhibit cycles and how well these cycles align with seizures. Second, we quantify how each of the measures performs under systematic spatial and temporal subsampling. Third, we determine how much these measures are correlated to one another in order to better understand whether they capture the same underlying phenomenon or not.

## 2 Methods

### 2.1 General overview

The methods section is organized as follows. First, the dataset is described along with the inclusion and exclusion criteria that were used to select the 23 patients from the Epilepsiae dataset Ihle et al. (2012). Second, we describe the preprocessing of the data prior to calculating the different metrics. Third, we systematically derive five network metrics that have been proposed as markers of seizure propensity (Table 1). We will describe these metrics in more detail, the motivation on using them and how each of them are calculated. These metrics are: Interictal Epileptiform Discharge Frequency (IEDF), Variance Measure (VM), Temporal Correlation Measure (TCM), Spatial Correlation Measure (SCM), Network Synchrony (NS). Fourth, we describe how the metrics and their cyclical occurrence are evaluated and compared against each other.

**TABLE 1:** Table of preprocessing steps, metrics that are evaluated, and criteria used for evaluation. Abbreviations: IEDF - Interictal Epileptiform Discharge Frequency, TCM - Temporal Correlation Measure, VM - Variance Measure, NS - Network Synchrony, SCM - Spatial Correlation Measure, PLV - Phase Locking Value, iEEG - invasive EEG.

	Preprocessing		Robustness		Evaluation criteria	
	Power band	iEEG band	Temporal sampling	Spatial sampling	Cycle	PLV
IEDF	N/A	N/A	yes	yes	yes	yes
TCM	yes	5 bands	yes	yes	yes	yes
VM	yes	5 bands	yes	yes	yes	yes
NS	yes	5 bands	yes	no	yes	yes
SCM	yes	5 bands	yes	no	yes	yes

### 2.2 Patient data

This study involved the analysis of multi-day invasive electroencephalographram (iEEG) recordings from 23 patients (12 female; 28 ± 13 years) diagnosed with focal epilepsy who were undergoing pre-surgical assessments at the Epilepsy Center of the University Hospital in Freiburg, Germany Ihle et al. (2012). The number of implanted electrodes was in the range of 40–120, iEEG data were recorded either at 256, 512, or 1024 Hz. Most of the datasets contained continuous, non-overlapping hour-long recordings over a period of 5–14 days for each patient. Few recordings were interrupted and thus under an hour, and were excluded from the analysis. Only a subsample of the entire Epilepsiae dataset consisting of 23 patients were used, where only the patients that varied in their anti-seizure medication (ASM) were included in the analysis Müller and Meisel (2023). The dataset had received ethics approval from the University of Freiburg’s ethics committee Ihle et al. (2012). All patients provided written informed consent, granting permission for their clinical data to be used and published for research purposes. The present study had also received approval from the local institutional review board (EK 92022019).

### 2.3 Preprocessing

The iEEG data was first notch filtered to remove power line noise at 50 Hz. A high pass filter was applied to remove the effects of slow drifts at 0.1 Hz, and a low pass filter removed the effects of noise and signals above 95 Hz (zero phase-lag 4th order butterworth). Next, since the iEEG recordings were all collected at different sampling frequencies, signals were downsampled to 256 Hz before the different metrics were calculated (using mne.resample via fft). An exception of this downsampling was for the preprocessing for the interictal epileptiform discharge (IED) algorithm, where we used a pretrained deep learning algorithm that required a sampling frequency of 200 Hz. More detail is given in Methods section 2.4.3. All of the processing steps were implemented using the MNE Python package that has been optimized for processing EEG data Gramfort et al. (2013).

The original dataset contained surface electrodes in addition to intracranial electrodes. However, the surface electrodes were omitted from the analysis because their data collection was often intermittent and did not cover the entire duration of the recordings. This exclusion was necessary to maintain consistency in the number of electrodes throughout the analysis period.

We also evaluated certain metrics individually for specific EEG frequency bands. For this purpose, we applied a bandpass filter at the following frequency ranges: 4–8 Hz (theta), 8–13 Hz (alpha), 13–30 Hz (beta), 55–95 Hz (gamma), 1–99 Hz (broadband). For our analyses, the broadband signal delivered essentially identical results to the delta signal (1–4 Hz), thus, to reduce redundant analyses, only results for the broadband signal are presented. The lower end of the gamma frequency range was truncated at 55 Hz, in order to further reduce the effects of the line noise at 50 Hz. In addition to quantifying metrics directly on the these frequency-filtered signals, we also performed analyses on power fluctuation timeseries in these frequency bands using eighth of a second non-overlapping windows Müller and Meisel (2023). In the final step of the preprocessing, we normalized the signal using sklearn preprocessing package over the entire hour before the subsequent analysis Pedregosa et al. (2011).

### 2.4 Evaluated metrics

All of the following metrics were first calculated on non-overlapping 2-min windows. Time windows preceding and following seizures, 5 minutes on either side, were excluded in the analysis. This was done to reduce the effects of seizures on these metrics as our goal was to track interictal activity. Seizure onset and offset times were labeled by expert clinicians. After excluding seizures, the metrics were averaged in time for each full hour, and, for single-electrode measures, they were also averaged across the electrodes in order to obtain a single value for each hour and patient. Random subsampling across electrodes and time was performed in order to generate the subsampled estimates to determine robustness.

#### 2.4.1 Temporal correlation measure

The TCM quantifies how quickly the information contained in a signal decays over time. As such, it is a measure of critical slowing down with maximal values near a critical point. For this reason, temporal correlations have been studied with respect to distance to seizure and have been shown to be a relevant measure in tracking cyclical changes to interictal activity as well as changes to ASM Maturana et al. (2020) Müller and Meisel (2023) Meisel (2020). More details of this methodology are provided in Müller and Meisel (2023). The TCM measure is one of the univariate, i.e., single-electrode, measures that is first calculated on each electrode separately. It is implemented by first calculating the autocorrelation function (ACF) of a signal: ACF(t)=f(n)⋆f(n+t) , where the star denotes the correlation function. In our case, this is calculated from the power fluctuations over a window that is 2 minutes long.

Following previous work Meisel (2020) Müller and Meisel (2023), for each iEEG channel, the time series of broadband gamma power fluctuations were obtained by calculating the power every 125 ms (Welch’s method, Hanning window) and applying the logarithm with base 10 to the timeseries. The signal is shifted once for each sample which determines the lower limit of the ACF resolution (0.125 s). Once the ACF is approximated, we estimate the time period it takes for the ACF to decrease to half the width at the maximum, also known as full-width-at-half maximum. This time period is then averaged spatially (i.e., across all electrodes) and temporally (i.e., across all 2-min windows in each hour) to obtain one value for the hourly period.

#### 2.4.2 Variance measure

A signal’s variance is similarly a measure of critical slowing down. As such it has also been suggested and studied with respect to seizure propensity Maturana et al. (2020) Meisel and Kuehn (2012). The VM is also a univariate measure that is calculated separately for each electrode. After preprocessing the signal, the variance is calculated independently for each 2-min interval: $\sigma^{2}=\frac{\sum {\left(x_{i}-\bar{x}\right)}^{2}}{n-1}$ . Once the variance is estimated for each electrode and for each 2-min segment, the average is obtained over the hourly interval and across all electrodes for each patient. To subsample, a random subset of either the electrodes or the intervals were included in the averaging.

#### 2.4.3 Interictal epileptiform discharge frequency

We used an openly available, validated deep learning software for automated IED detection Quon et al. (2022). The algorithm first identifies candidate IEDs using a triangle convolution and thresholding. Around these candidate IEDs, a spectrogram is calculated and is fed into a classifier to determine if the segment contains an IED or not. After applying the detection algorithm, we determined the number of IEDs per channel and minute in each 2-min interval by dividing the total number of IEDs during that interval in all electrodes by the number of electrodes. Only channels that were in the seizure onset zone were considered, as other channels had few or no IEDs. The seizure onset zones were predetermined by clinicians.

#### 2.4.4 Network synchrony

Insights into cortical activity from computational modeling, rodent and human EEG have pointed to the ability of global synchronization measures to characterize physiological cortical dynamics Meisel and Kuehn (2012) Meisel et al. (2015b) Meisel (2016). It has been shown that phase synchronization of ongoing cortical activity can been linked to the stability of the neural cortex. NS is a multivariate network measure, and is calculated across all electrodes simultaneously. We utilize the methodology described in detail in Meisel et al. (2015b) to estimate the NS across electrodes. In short, to quantify the NS, we first calculate the Hilbert transform to derive the instantaneous phase of the signal in each electrode. We then average across all the phases across all electrodes E in the complex domain: $NS=Re\left(\frac{1}{E}\sum_{\theta_{e}}^{\forall E}e^{i*\theta_{e}}\right)$ . If all phases are aligned then the NS is equal to 1, and for a completely asynchronous process the NS would be 0. The NS is calculated for each time point within a 2-min interval and then averaged across the interval to produce an estimate of NS for the 2-min window. We then average across all or a subsample of all 2-min periods over the hour to estimate the hourly rate and the subsampled hourly rate.

#### 2.4.5 Spatial correlation measure

The hallmark of a dynamical system in the critical regime is the presence of long-range correlations which, in spatial systems, decay as a function of distance Müller and Meisel (2023). In Müller and Meisel (2023) spatial correlations were shown to be sensitive to changes of ASM levels as well as to track circadian rhythms. For this measure, we follow the methodology described in Müller and Meisel (2023), where the correlation between each pairs of electrodes is first computed over a 2-min window. To measure how spatially correlated all the signals are from each other, we take the average the correlation of all electrodes that are between 10 and 80 mm from each other. The interval was chosen as most of the patients had electrodes within these distances. Once the measure has been estimated for each 2-min segment, the values are all averaged together or subsampled to produce an estimate of the hourly rate.

#### 2.4.6 Null model

In addition to the five metrics described, we also constructed a null model exhibiting a simple 24-h cycle modelled by a simple cosine function. This was used to evaluate if the metrics contained more information than just using an hourly clock to quantify interictal activity without using iEEG data. We utilized this null model to also correlate against all the other signals to see how well they track 24-h rhythms. We refer to this model as the Cosine Daily Function (CDF).

### 2.5 Evaluation criteria

Our goal was to determine whether and how the five metrics of seizure propensity are related to each other, whether they exhibit 24-h cycles, and to determine the effects of spatial and temporal subsampling on these measures.

#### 2.5.1 Periodogram

The periodogram from hourly timeseries in each patient was used to assess the power of cycles, and 24-h cycles in particular. The hourly timeseries were first interpolated in case of missing hours, removed from outliers that were greater than three standard deviations from the mean, and then converted into periodograms using the Welch’s method Baud et al. (2018). In order to test if the peak at 24 h was significant, we calculated the average periodogram across all patients, and fitted a red noise spectrum $\frac{1}{f^{\alpha }}$ that matched the average frequency distribution. By determining the variance from the average frequency distribution, we then simulated 10,000 periodograms by adding the mean and variance, and measured how many times the randomly generated signal would result in a peak larger than the peak measured at 24 h. From this we estimated the *p*-value and significance at the 24-h peak in each patient. We then determined how many significant 24-h cycles were detected across our cohort.

#### 2.5.2 Phase locking value

The Phase Locking Value (PLV) was used to evaluate how well seizures aligned with the interictal measures. The PLV is calculated by averaging the phases when the seizures occur in the complex plane. In previous publications, this value was determined by using a wavelet transform on the metric timeseries and decomposing the activity to daily, weekly, monthly and even yearly cycles and then averaging the phase for each of these cycles individually Baud et al. (2018) Karoly et al. (2021), Stirling et al. (2021). Since our recordings are at most 18 days and we were mostly interested in the 24-h rhythm, we filtered the timeseries using a bandpass filter between 2 and 30 h using a zero-phase lag filter of a single order. We subsequently performed a Hilbert transform to estimate the instantaneous phase of the signal over time and calculated the PLV by summing the phases at which the seizures S occurred: $PLV=Re\left(\frac{1}{S}\sum_{\theta_{s}}^{\forall S}e^{i*\theta_{s}}\right)$ . The PLV is a measure between 0 and 1 where the closer to 1 means the more the phases at which seizures occur are similar.

## 3 Results

### 3.1 Tracking cycles

**FIGURE 1:**
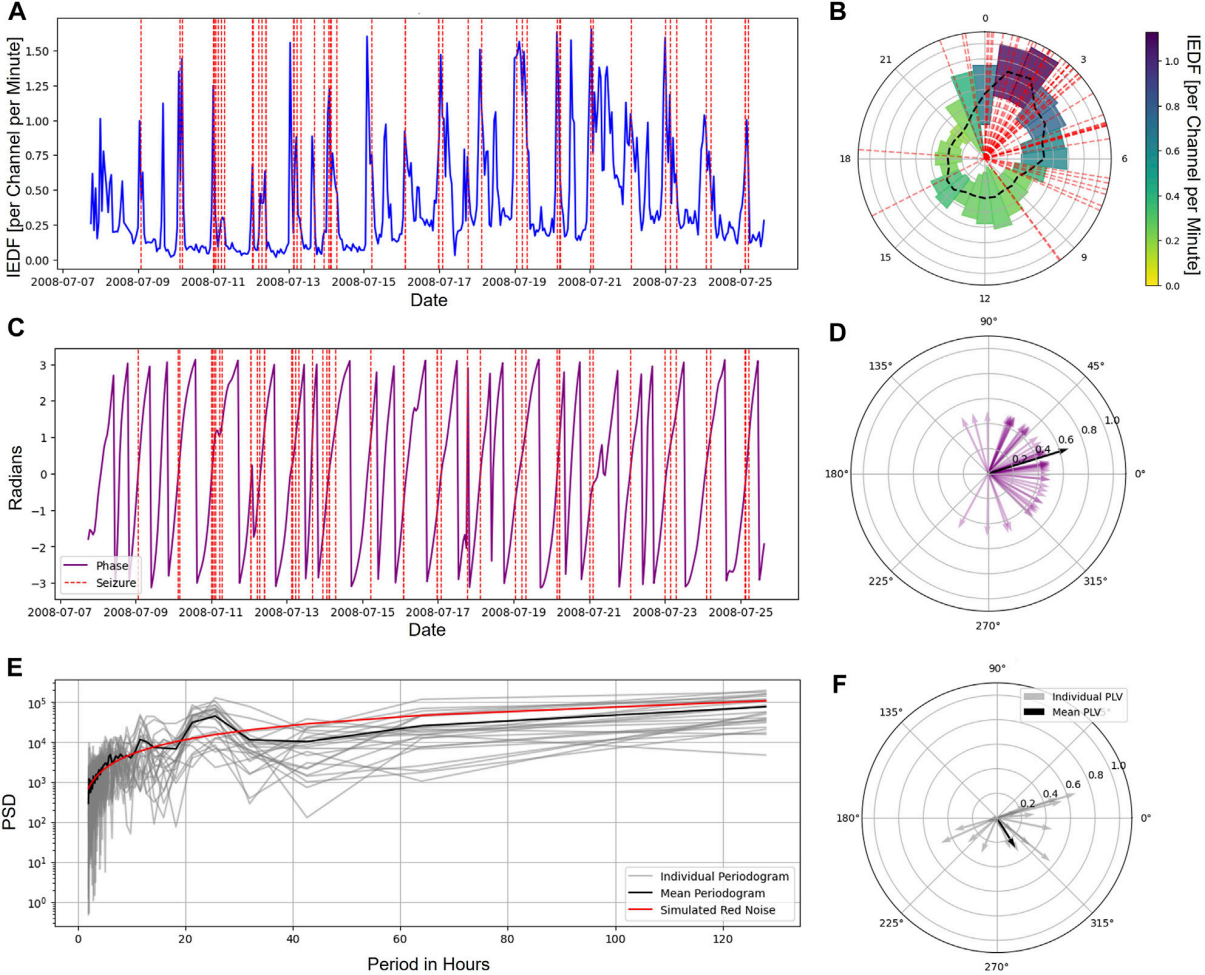
Cycles in interictal iEEG markers and their relationship to seizures. **(A)**, Average IEDF timeseries tracked over the span of multiple days for a sample patient. The vertical red lines represent seizures. **(B)**, IEDF plotted on a 24-h clock with the mean (black dashed) and variance (width) estimated for each hour separately for a sample patient. The seizures (shown in red dashed line) and high IEDF coincide during the hours after midnight. **(C)**, IEDF phase timeseries for a sample patient. **(D)**, The alignment of the phase of each seizure (purple) as well as the PLV across all seizures for the sample patient (black). **(E)**, Periodograms for each individual patient (grey) and the group mean (black). The best fitted red noise is plotted in red, significant peaks were found at 12 and 24 h for the mean plot. **(F)**, The PLVs for individual patients as well as mean for the group. Abbreviations: IEDF - Interictal Epileptiform Discharge Frequency; PLV - Phase Locking Value.

We first characterized the five metrics with respect to exhibiting cycles and how well these cycles aligned with seizures. Figure 1 shows the timeseries of a single metric (interictal epileptiform discharge frequency, IEDF) over the course of the whole monitoring period. The respective versions for the other four metrics are plotted in Supplementary Figures S4–7. The IEDF exhibited a 24-h pattern in this patient, as captured by the clock plot and the periodogram (Figures 1B, E). For this particular patient, seizures preferentially occurred during the increasing phase in IEDF during the first few hours after midnight. This relationship was captured by the phase locking of seizures to a certain phase of IEDF where the seizures mostly occur close to zero degrees (Figure 1D).

Following previous work, we were primarily interested in TCM and SCM obtained from the gamma power timeseries Meisel (2020) Müller and Meisel (2023), NS from the filtered gamma signal Meisel et al. (2015b) Meisel (2016), and VM calculated from the broadband signal. Under full spatial and temporal sampling of the data, we observed that 15 of 23 patients exhibited significant 24-h cycles in IEDF (65%), 18 in TCM (78%), 19 in VM (74%), 19 in SCM (83%) and 18 in NS (78%). Thus, all five metrics exhibited a strong 24-h cycle in the majority of patients, with the lowest number of patients with cycles in IEDF. Next, we evaluated how well each metric was phase locked to seizures by calculating the group PLV. Besides exhibiting 24-h cycles in the most patients, the metrics SCM, NS and VM also exhibited the highest PLV values (0.34 ± 0.24, 0.38 ± 0.24 and 0.37 ± 0.21, respectively). IEDF and TCM exhibited overall smaller PLV values (0.29 ± 0.18 and 0.26 ± 0.23, respectively). As a control, CDF provided a PLV of 0.18 ± 0.21.

### 3.2 Robustness under subsampling

**FIGURE 2:**
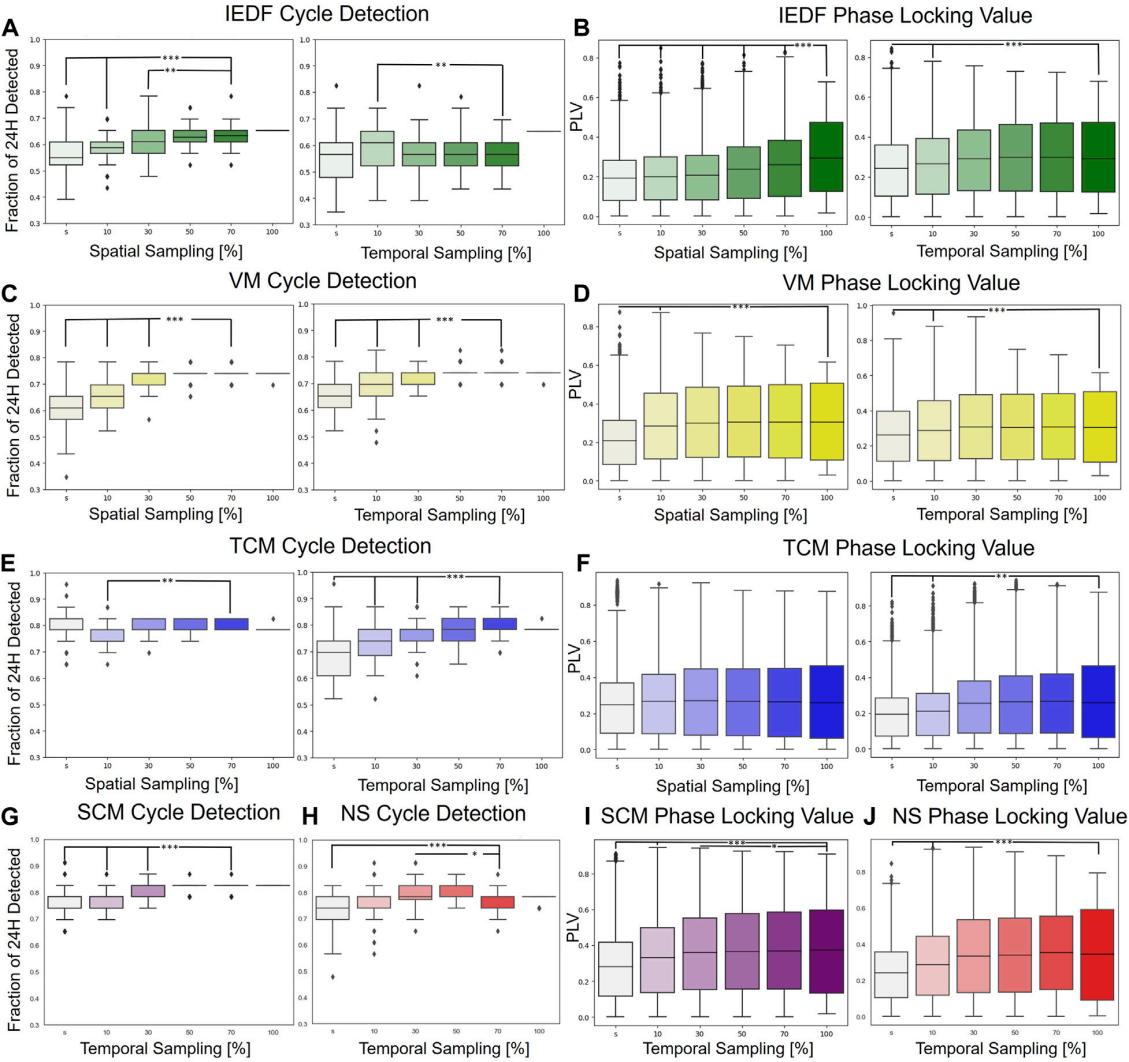
Robustness under spatial and temporal subsampling with regards to detection of 24-h cycles and phase locking to seizures. **(A)**, IEDF cycle detection under spatial (left) and temporal subsampling (right). **(B)**, IEDF PLV under subsampling. **(C, D)**, VM under subsampling. **(E, F)**, TCM under subsampling. **(G–J)**, cycle detection and PLV for SCM (purple) and NS (red) under temporal subsampling. These two multi-electrode measures were only subsampled temporally. The small “s” represents a single electrode or a single 2 min segment. *p < 0.05, **p < 0.01, and ***p < 0.001, measured against 100 percent for the PLV and 70 percent for the cycle detection. Abbreviations: IEDF - Interictal Epileptiform Discharge Frequency, TCM - Temporal Correlation Measure, VM - Variance Measure, NS - Network Synchrony, SCM - Spatial Correlation Measure.

Next, we investigated how strongly these measures exhibited cycles with relationship to seizures under spatial and temporal subsampling. This analysis was motivated by the fact that implantable neurorecorders are often constrained in their ability to record spatially and temporally. For the single-electrode metrics (IEDF, TCM, VM) both the spatial and temporal subsampling were performed, while for the multi-electrode metrics (SCM and NS) only the temporal subsampling was estimated. In Figure 2 the left half represents the number of significant cycles detected while the right half represents PLVs under subsampling. The small “s” in the Figure denote a single electrode or a single 2 min interval.

Most measures were highly robust under subsampling as indicated by few significant differences between using the full spatial and temporal interval vs. just using just 10 percent of the original total electrodes/time. Even if the differences were significant, they were not large in terms of absolute change. The univariate metrics, in particular VM and TCM, were especially robust, and even performed robustly for a single electrode. One of the few exceptions was the spatial subsampling of the IEDF which performed significantly worse for the PLV measure. This is likely because of the omission of electrodes in the seizure onset zone that contained the most IED under subsampling. The results for the different EEG bands, δ,θ,α,β,γ and preprocessing methods are given in Supplementary Figure S8 for the metrics described in Table 1.

### 3.3 Comparison of correlations between metrics

**FIGURE 3:**
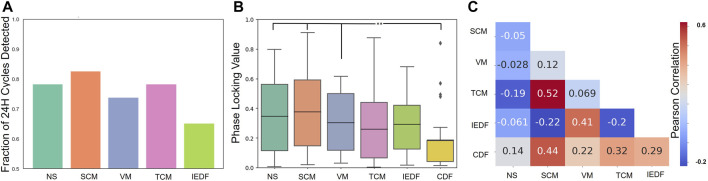
Comparison of correlations between metrics. **(A)**, Cycle detection across metrics, i.e., how many significant 24-h cycles were detected across 23 patients for each metric. **(B)**, Phase locking value across metrics. **(C)**, Relationship between pairs of metrics. The Pearson correlation calculated from hourly timeseries between pairs of metrics is displayed. In addition, the metrics are compared to how much they correlate to the CDF. *p < 0.05, **p < 0.01, ***p < 0.001. Abbreviations: IEDF - Interictal Epileptiform Discharge Frequency, TCM - Temporal Correlation Measure, VM - Variance Measure, NS - Network Synchrony, SCM - Spatial Correlation Measure, PLV - Phase Locking Value, CDF - Cosine Daily Function.

Finally, we determined how the metrics were correlated to one another. Figure 3 A, B show the summary for all metrics in terms of exhibiting 24-h cycles and seizure alignment with these cycles. The average Pearson correlation between pairs of metrics is displayed in Figure 3C. Apart from the control (CDF), two pairs of metrics exhibited notable positive correlations: SCM and TCM (Pearson correlation of 0.52) as well as VM and IEDF (Pearson correlation of 0.41). TCM and SCM both measure long-range correlations in time and space, respectively. Within the framework of critical transitions a close correlation between the two is expected Müller and Meisel (2023). The correlation between IEDF and VM and their ability to track seizures is in line with observations from previous studies Maturana et al. (2020). Conversely, IEDF was weakly anticorrelated to both TCM and SCM.

## 4 Discussion

Several iEEG metrics have been proposed and shown to track evolving epileptic brain networks and the changing seizure risk levels. Such metrics have important implications for seizure risk assessment and forecasting methods. However, it is currently not fully understood whether these different metrics measure the same underlying dynamical changes and how they perform under spatial and temporal subsampling. Our results show that most of these measures appear to change gradually in time and are robust to temporal and spatial subsampling. The univariate metrics, in particular VM and TCM, were especially robust, and even performed robustly for a single electrode. While all five measures exhibited 24-h cycles in the majority of patients, the strength of such cycles varied between measures with the least pronounced 24-h cycles exhibited in IEDF. Based on evaluations on 24-h cycles, SCM, NS and VM exhibited the highest phase locking with seizures while IEDF exhibited lowest PLV values. While it remains to be determined whether these observations also hold for longer, multi-dien cycles, our results provide some mechanistic insights on seizure propensity dynamics and may guide the choice of appropriate metrics for long-term prediction of seizure risk periods.

### 4.1 Robustness under subsampling

All of the metrics were calculated using 2 minute intervals and exhibited gradual change over the course of days and over 24-h cycles. Systematic temporal subsampling demonstrated that most metrics only really needed a snapshot of the hour to robustly track the changes to interictal activity. For example, subsampling to 2 minutes per hour reduced the number of cycles detected to ninety percent of the original cycles detected over the entire 60 min period for most metrics. For IEDF this number even remained unchanged.

The metrics were also relatively invariant to which region from the brain they were sampled from. A particular exception was IEDF. IEDs are more prevalent in the seizure onset zone and therefore performed worse when spatially subsampled, which naturally limited sampling from the seizure onset zone. The other metrics were less affected by spatial subsampling, with TCM not having any significant difference if being calculated using all electrodes or a single electrode.

Collectively, these observations suggest that the intrinsic properties of the neural system can be efficiently tracked with interictal cycles also under subsampling. Thus, for future implanted devices, especially those that have fewer electrodes, tracking interictal cycles with respect to seizure risk periods may still be feasible Haneef et al. (2022).

### 4.2 Relationship between cycles and seizures

Interictal cycles of iEEG metrics have been linked to seizure occurrence Baud et al. (2018) Stirling et al. (2021) Maturana et al. (2020). While different metrics may exhibit cycles, we here aimed to evaluate how strongly exactly different metrics were locked to seizure occurrence. Based on evaluations over 24-h cycles, SCM, NS, and VM demonstrated the strongest clustering with seizures. In contrast, IEDF, commonly used for cycle detection, showed the lowest phase locking value (PLV). These observations suggest further testing to determine if these measures also correlate strongly with seizures in longer-term recordings and across multi-dien cycles. VM has already been tested in this context demonstrating strong capability to track and forecast seizure risk Maturana et al. (2020). The further investigation of metrics like SCM, NS and VM is furthermore supported by our observation that these metrics also exhibited stronger 24-h cycles in more patients that compared to IEDF.

### 4.3 Comparison of correlation between metrics

For the understanding of seizure dynamics and choice of metrics to monitor it is an important question whether all metrics measured the same underlying dynamical phenomenon or whether they were (at least partially) complementary to each other. All of the metrics’ hourly timeseries exhibited high correlation with the CDF indicating that they measure some 24-h rhythmicity. However, they also seem to have unique information that is specific to each metric. Notably, the CDF performed significantly worse in PLV than to other measures, suggesting that it is not simply the time of day that is informative to track cycles.

Measures derived from criticality theory (NS, SCM, TCM, VM) generally exhibited stronger phase locking to seizures than IEDF or the control CDF. Albeit only indirect, these observations provide further support that critical dynamics and critical slowing down may potentially be a useful framework to characterize the state of cortical networks, including their transition to seizures Meisel and Kuehn (2012) Meisel et al. (2015b) Meisel (2016) Maturana et al. (2020) Meisel and Loddenkemper (2020) Müller and Meisel (2023). The relationship between metrics is also consistent with previous understanding of these processes. SCM and TCM, the spatial and temporal correlation metrics, have a very large positive correlation Müller and Meisel (2023). This is a phenomenon that occurs closer to criticality prior to the system transitioning to a more unstable regime Meisel and Loddenkemper (2020) Scheffer et al. (2009). The VM and the IEDF measure also seem to be related which is similar to what has been shown when comparing these metrics for epileptic cycles Maturana et al. (2020). The two network measures such as SCM are the closest measure to circadian rhythms, potentially suggesting that they are the most sensitive to changes between sleep and awake EEG recordings.

## 5 Limitations

The main limitation of this work is that recordings analyzed were over a relatively short time frame of only several days. Cycles that are on order of days or the much longer multi-dien cycles on the order of weeks/months could naturally not be analyzed. However, our results point to specific metrics that could consequently be investigated on longer recordings in future work. Furthermore, when comparing our PLVs and the number of 24-h cycles detected, our results seem to be comparable to results from other recent studies. Specifically, the mean value of the PLV for NS (slightly less than 0.4), is comparable to those of previous literature, while the other metrics have a mean PLV similar to what was previously published Baud et al. (2018) Stirling et al. (2021) Maturana et al. (2020). For the 24-h cycles we were able to detect them in about 83 percent of the patients. This is also comparable to previous estimates of cycle detection that was around 70 percent Baud et al. (2018).

## 6 Conclusion

In this paper we have shown that interictal cycle detection and its relationship to seizure risk periods are quite robust under spatial and temporal subsampling. While some of the measures are similar to each other, they also appear to capture (at least partially) complementary information. Measures derived from criticality show stronger capability to capture cycles locked to seizures in comparison to more traditional markers such as IEDF. Future research is needed to validate these results on longer data. Our results may help to better choose appropriate metrics for implantable devices to detect cycles and forecast seizures in the future.

## Data Availability

The data analyzed in this study is subject to the following licenses/restrictions: Data is available from the public Epilepsiae database, and can be accessed by request. Requests to access these datasets should be directed to https://epilepsy-database.eu/.
