# Supplementary figures and images for: Critical dynamics and interictal epileptiform discharge: a comparative analysis with respect to tracking seizure risk cycles
Source: Front Netw Physiol. 2024 Jul 9;4:1420217. doi: 10.3389/fnetp.2024.1420217 (PMC11263032; doi:10.3389/fnetp.2024.1420217)

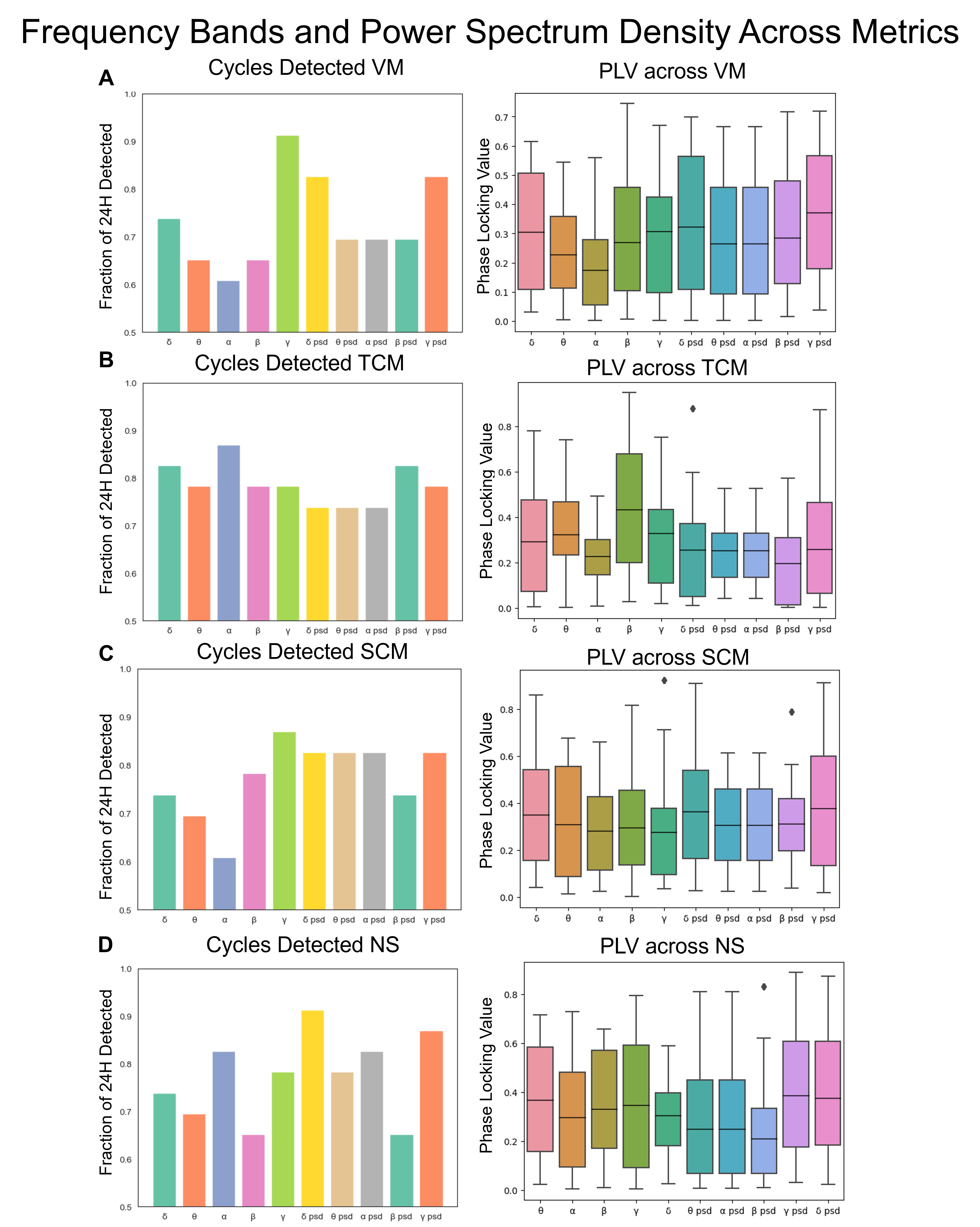

Supplement: Supplementary file 1 [file Image5.PNG]

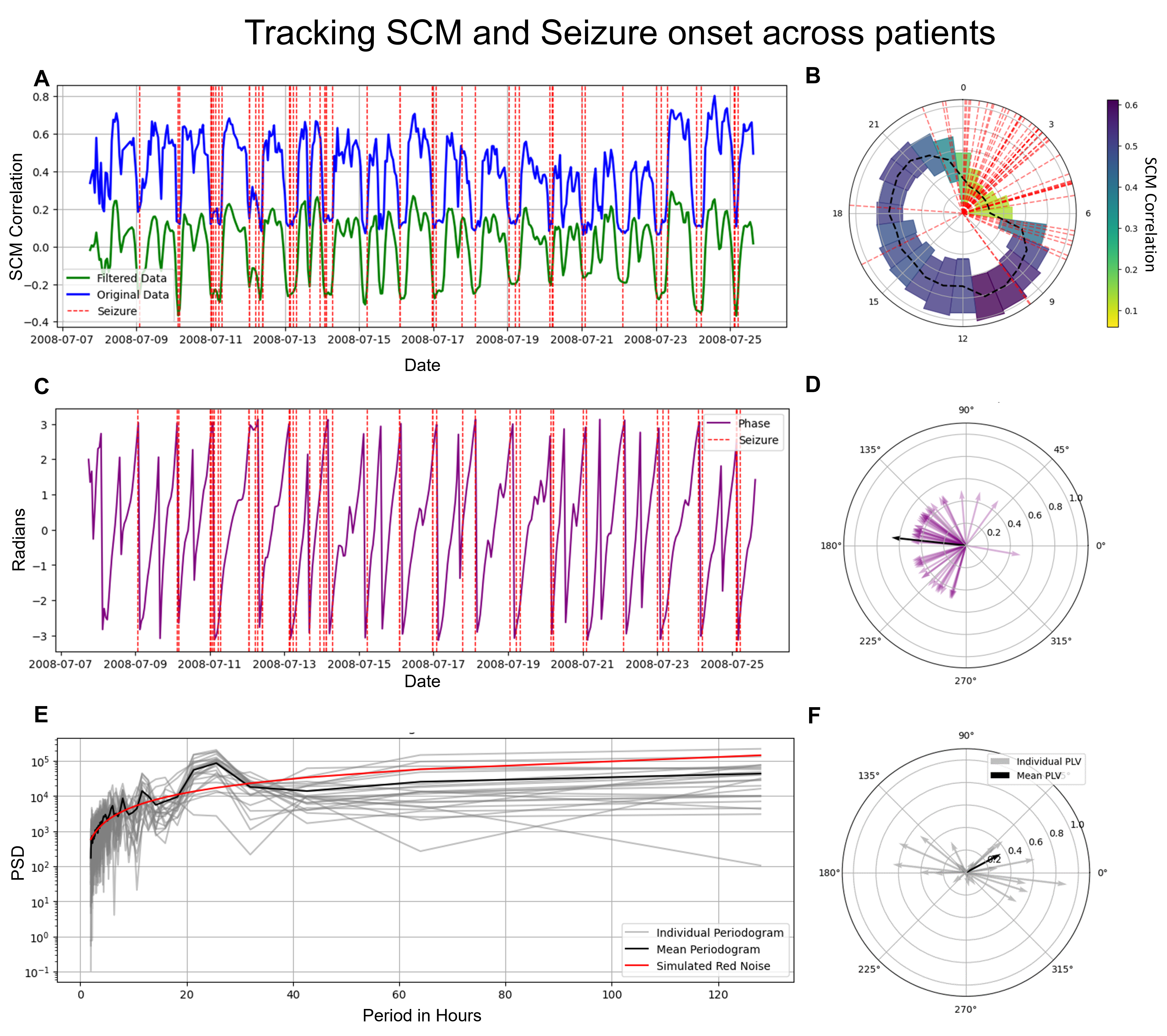

Supplement: Supplementary file 2 [file Image4.PNG]

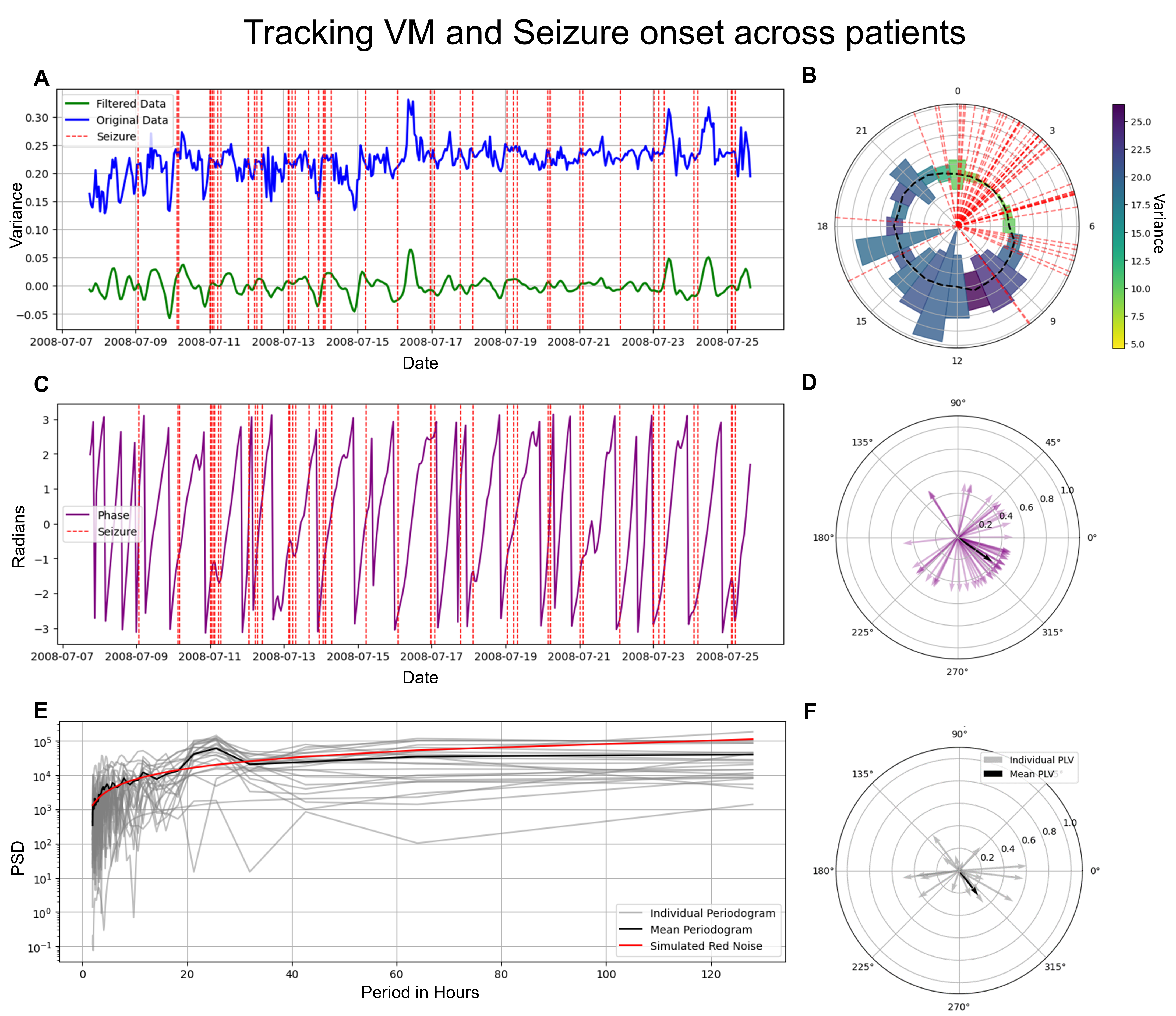

Supplement: Supplementary file 3 [file Image2.PNG]

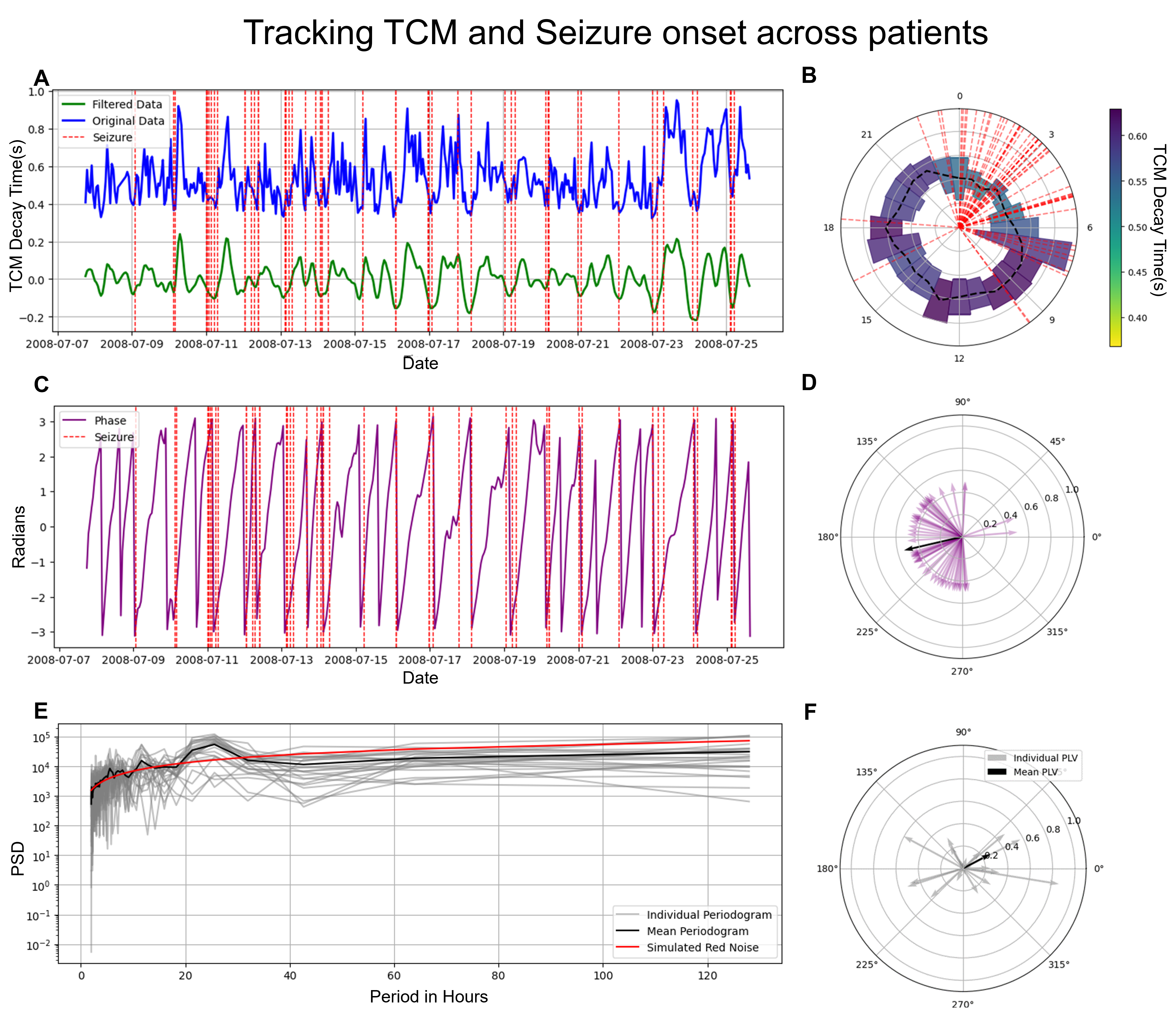

Supplement: Supplementary file 4 [file Image1.PNG]

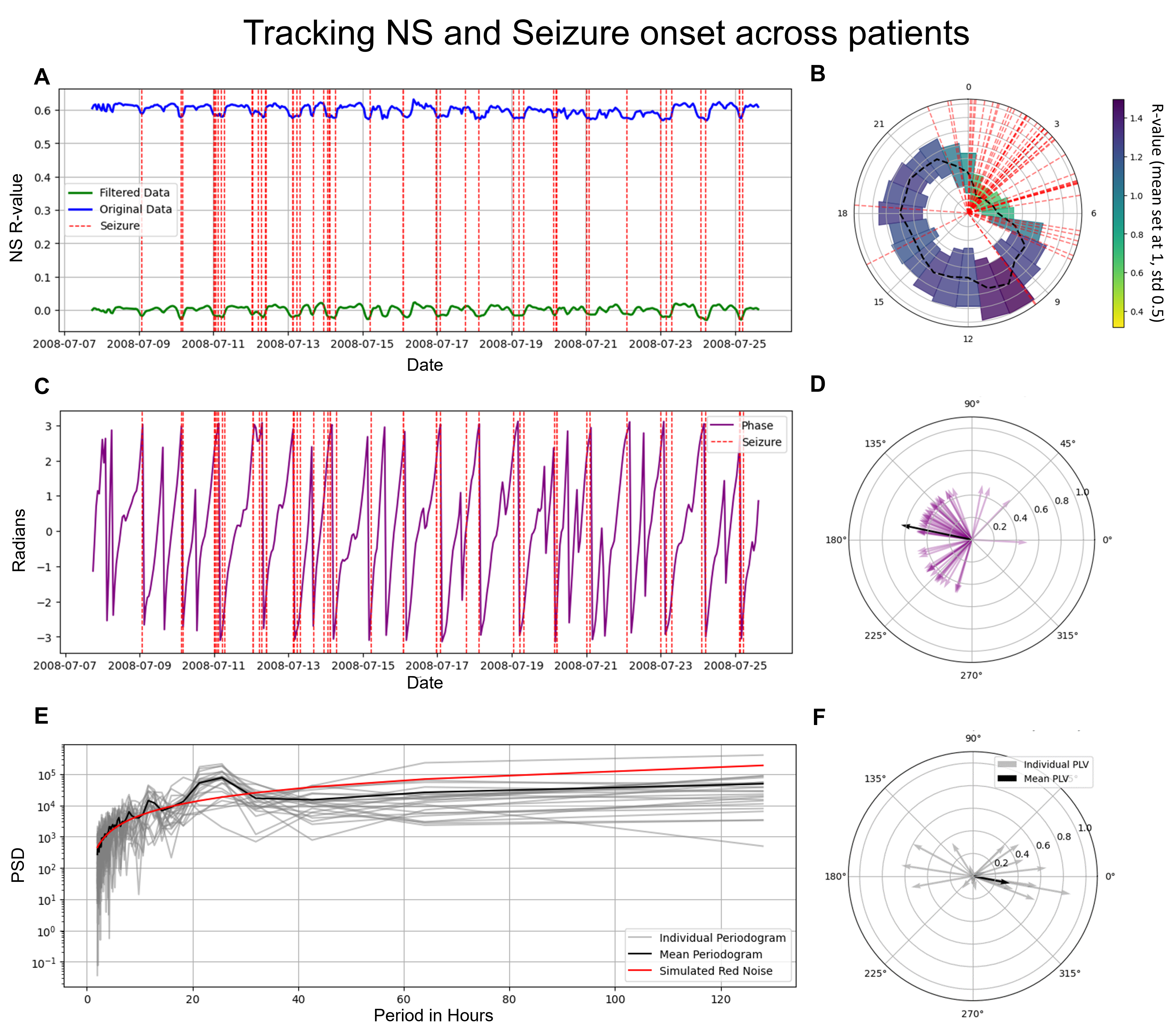

Supplement: Supplementary file 5 [file Image3.PNG]
